# Recurrent Pleomorphic Adenoma of the Palate: A Case Report

**DOI:** 10.3390/clinpract16090168

**Published:** 2026-09-10

**Authors:** Evangelos Kostares, Stavroula Diamantopoulou, Ourania Schoinohoriti, Georgia Kostare, Ioli Artopoulou, Georgios Mitsopoulos, Christos Perisanidis

**Affiliations:** 1 Department of Oral and Maxillofacial Surgery, Dental School, National and Kapodistrian University of Athens, 115 27 Athens, Greeceour.schoino@gmail.com (O.S.);; 2 Department of Microbiology, Medical School, National and Kapodistrian University of Athens, 115 27 Athens, Greece; 3 Department of Prosthodontics, Dental School, National and Kapodistrian University of Athens, 115 27 Athens, Greece; 4 Department of Oral and Maxillofacial Surgery, 401 General Military Hospital of Athens, 115 25 Athens, Greece

**Keywords:** pleomorphic adenoma, recurrent, head and neck, palate, minor salivary glands, case report

## Abstract

**Background:** Pleomorphic adenoma is the most common benign salivary-gland neoplasm and frequently affects the minor salivary glands of the palate. Although surgical excision is generally curative, incomplete encapsulation, pseudopod-like extensions, and microscopic tumor deposits may contribute to recurrence, which can occur many years after initial treatment. Recurrent palatal pleomorphic adenoma is uncommon and may present diagnostic and reconstructive challenges. **Case Presentation:** A 64-year-old man was referred with a painless, slowly enlarging recurrent mass of the right posterior hard palate, six years after excision of a pleomorphic adenoma at another institution. Magnetic resonance imaging and contrast-enhanced computed tomography demonstrated a well-defined enhancing palatal lesion measuring approximately 2.1 × 1.9 × 1.9 cm on MRI and 1.8 × 2.2 × 1.9 on CT, without adjacent maxillary bone destruction or cervical lymphadenopathy. An incisional biopsy was consistent with pleomorphic adenoma. Wide local excision was performed, including the tumor, surrounding palatal soft tissue, and underlying periosteum. The resulting defect was reconstructed using a facial artery musculomucosal flap. The postoperative course was uneventful, with satisfactory flap healing and preservation of speech and swallowing. At six months of follow-up, there was no clinical evidence of recurrence or functional impairment. **Conclusions:** Recurrent pleomorphic adenoma of the palate may develop several years after primary treatment. Complete surgical excision with tumor-free margins and appropriate reconstruction is essential. Long-term clinical follow-up is recommended because late recurrence and, rarely, malignant transformation may occur.

## 1. Introduction

Pleomorphic adenoma is the most common benign tumor of the salivary glands and frequently arises from minor salivary glands, with nearly half of intraoral cases occurring in the palate. Clinically, palatal pleomorphic adenoma typically presents as a slow-growing, painless, firm submucosal nodule covered by intact mucosa and usually demonstrates a favorable prognosis after surgical excision. However, incomplete encapsulation and microscopic extensions beyond the apparent tumor margin may predispose to local recurrence, which may occur many years after primary treatment [1,2,3,4]. Histopathological studies have further demonstrated that pleomorphic adenomas, particularly those arising from minor salivary glands, may exhibit capsular infiltration, pseudopod-like extensions, and satellite nodules, features that may complicate complete surgical removal and contribute to subsequent recurrence [2,3,4,5,6].

Recurrent pleomorphic adenoma of the palate represents a rare but clinically significant condition. Available evidence indicates that recurrence may develop decades after initial surgery, with increasing odds of detection at 10 and 20 years of follow-up. Although malignant transformation is uncommon, it remains an important consideration, particularly in long-standing pleomorphic adenoma. Malignant transformation has been documented in recurrent lesions and in pleomorphic adenomas arising from minor salivary glands [7,8,9,10]. More recent longitudinal evidence suggests that prolonged tumor duration may play a more important role in malignant transformation than recurrence itself, and recurrence should therefore not necessarily be regarded as an independent causal risk factor. Nevertheless, the possibility of malignant transformation further reinforces the importance of long-term surveillance in patients with recurrent disease [6,7].

Histopathologically, pleomorphic adenoma demonstrates epithelial and myoepithelial components within a variable stromal background. Myoepithelial-predominant or monomorphic variants may represent early or limited morphologic expressions of the same neoplastic spectrum, occasionally preceding the development of classic mixed-tumor morphology in recurrent lesions [1].

The surgical management of palatal pleomorphic adenoma requires complete tumor removal while avoiding unnecessary resection of uninvolved adjacent structures. Recent palate-specific studies have highlighted substantial variability in pseudocapsule thickness, pseudopodial extensions, and satellite nodules and have emphasized the importance of excising the tumor with an adequate surrounding soft-tissue margin [2,3,4,5,6]. Excision of the underlying periosteum may provide an appropriate deep surgical boundary when there is no evidence of osseous invasion, whereas resection of the palatal or maxillary bone should be determined by radiological and intraoperative evidence of bone involvement [2,3]. Avoidance of capsular disruption is also important because intraoperative tumor rupture has been associated with recurrence [8,11].

Given the rarity of recurrent palatal pleomorphic adenoma and the limited literature describing both its oncological and reconstructive management, detailed reporting of individual cases remains clinically relevant. We present a case of recurrent pleomorphic adenoma of the hard palate, occurring approximately six years after primary excision and highlighting the diagnostic evaluation, rationale for surgical management with preservation of the uninvolved palatal bone, and immediate reconstruction of the resulting soft-tissue defect with a facial artery musculomucosal flap.

## 2. Case Presentation

This case report was prepared in accordance with the CARE guidelines. The CARE checklist has been completed and is provided as Supplementary Materials (Supplementary Table S1).

A 64-year-old man was referred to our department for evaluation and treatment of a recurrent right palatal tumor. His medical history was unremarkable apart from mild benign prostatic hyperplasia. No cervical lymphadenopathy was identified.

The patient had initially presented in 2020 at another institution with a painless, slowly enlarging swelling of the right palate and underwent surgical excision. Histopathological examination was reported as a pleomorphic adenoma. According to the available previous pathological information, the lesion measured 1.5 × 1.0 × 0.7 cm and contained epithelial and myoepithelial cells arranged in duct-like and trabecular structures within hyalinized and focally myxoid stroma. Squamous metaplasia and small mucinous deposits were present, whereas significant cytological atypia, increased mitotic activity, or evidence of malignancy was absent.

Approximately six years after the initial operation, the patient was referred to our department for the first time because of a recurrent, slowly enlarging, painless swelling of the right posterior palate. Clinical examination demonstrated a well-circumscribed, firm, sessile submucosal mass covered predominantly by intact mucosa. There was no clinical evidence of regional lymph-node involvement.

**Figure 1:**
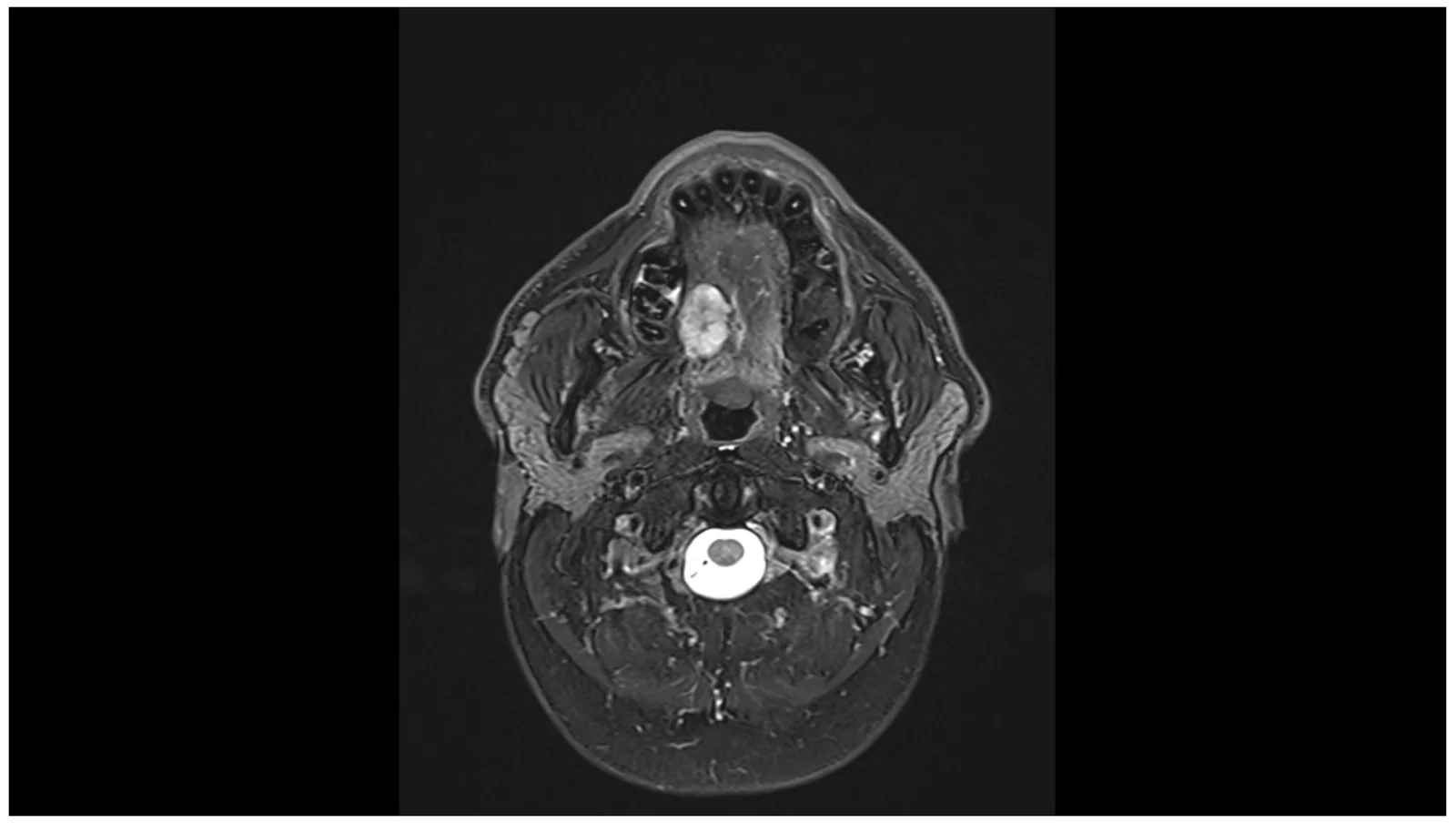
Axial fat-suppressed T2-weighted image demonstrates a well-circumscribed, heterogeneously hyperintense lesion arising from the right posterior palate.

**Figure 2:**
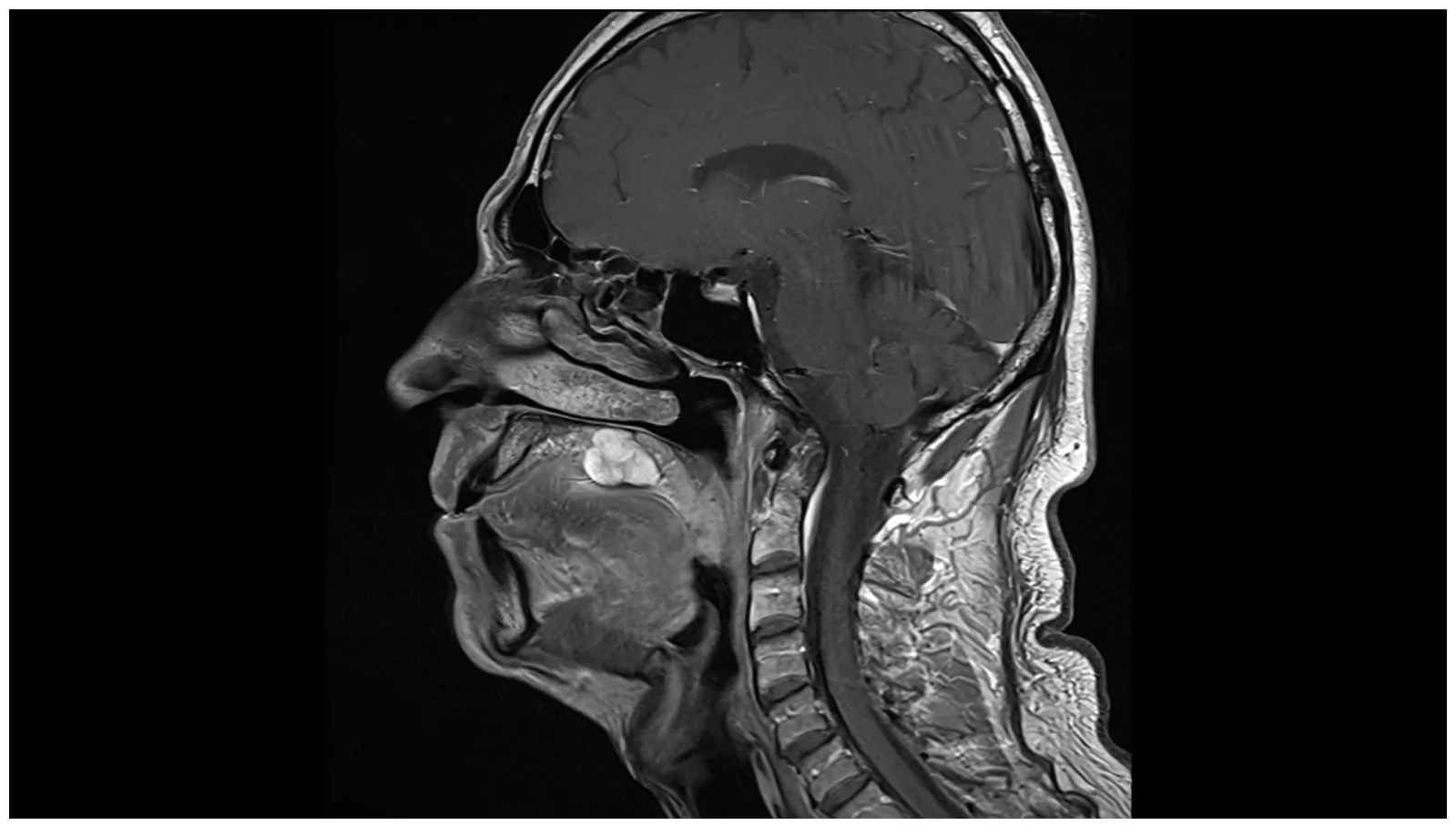
Sagittal contrast-enhanced T1-weighted image demonstrates marked heterogeneous enhancement of the palatal lesion.

Magnetic resonance imaging performed on 22 January 2026 revealed a solid lesion arising from the right lateral aspect of the right posterior hard palate. The lesion exhibited low signal intensity on T1-weighted sequences, high signal intensity on T2-weighted sequences, and marked enhancement following intravenous contrast administration. It measured approximately 2.1 × 1.9 cm in the axial plane and 1.9 cm craniocaudally. There were no enlarged or morphologically suspicious cervical lymph nodes. Representative magnetic resonance images are shown in Figure 1 and Figure 2.

Contrast-enhanced computed tomography performed on 24 January 2026 demonstrated a well-defined soft-tissue lesion of the right posterior hard palate measuring approximately 1.8 × 2.2 × 1.9 cm. The lesion enhanced after contrast administration but caused no erosion, destruction, or other involvement of the adjacent maxillary bone. The imaging findings were therefore consistent with a localized tumor without radiological evidence of osseous invasion.

Given the patient’s previous histopathological diagnosis, recurrent pleomorphic adenoma was the leading diagnostic consideration. The differential diagnosis included malignant transformation within a recurrent pleomorphic adenoma. The slow, painless clinical progression, well-circumscribed imaging appearance, absence of osseous destruction or suspicious cervical lymphadenopathy, and subsequent incisional-biopsy findings favored recurrent pleomorphic adenoma.

**Figure 3:**
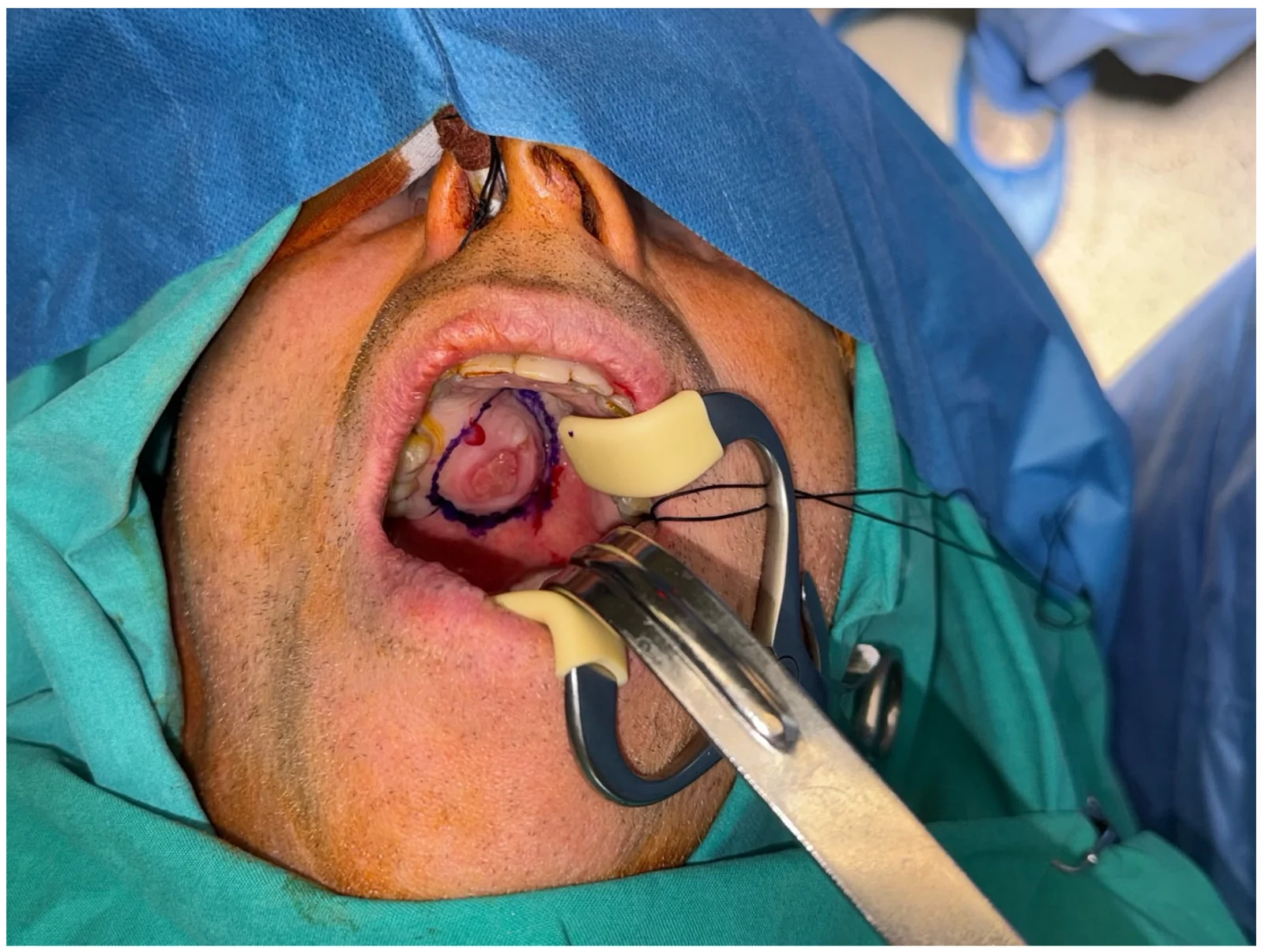
Intraoperative view of the recurrent pleomorphic adenoma of the right posterior hard palate. The clinical margins of the well-circumscribed palatal lesion were marked before surgical excision.

**Figure 4:**
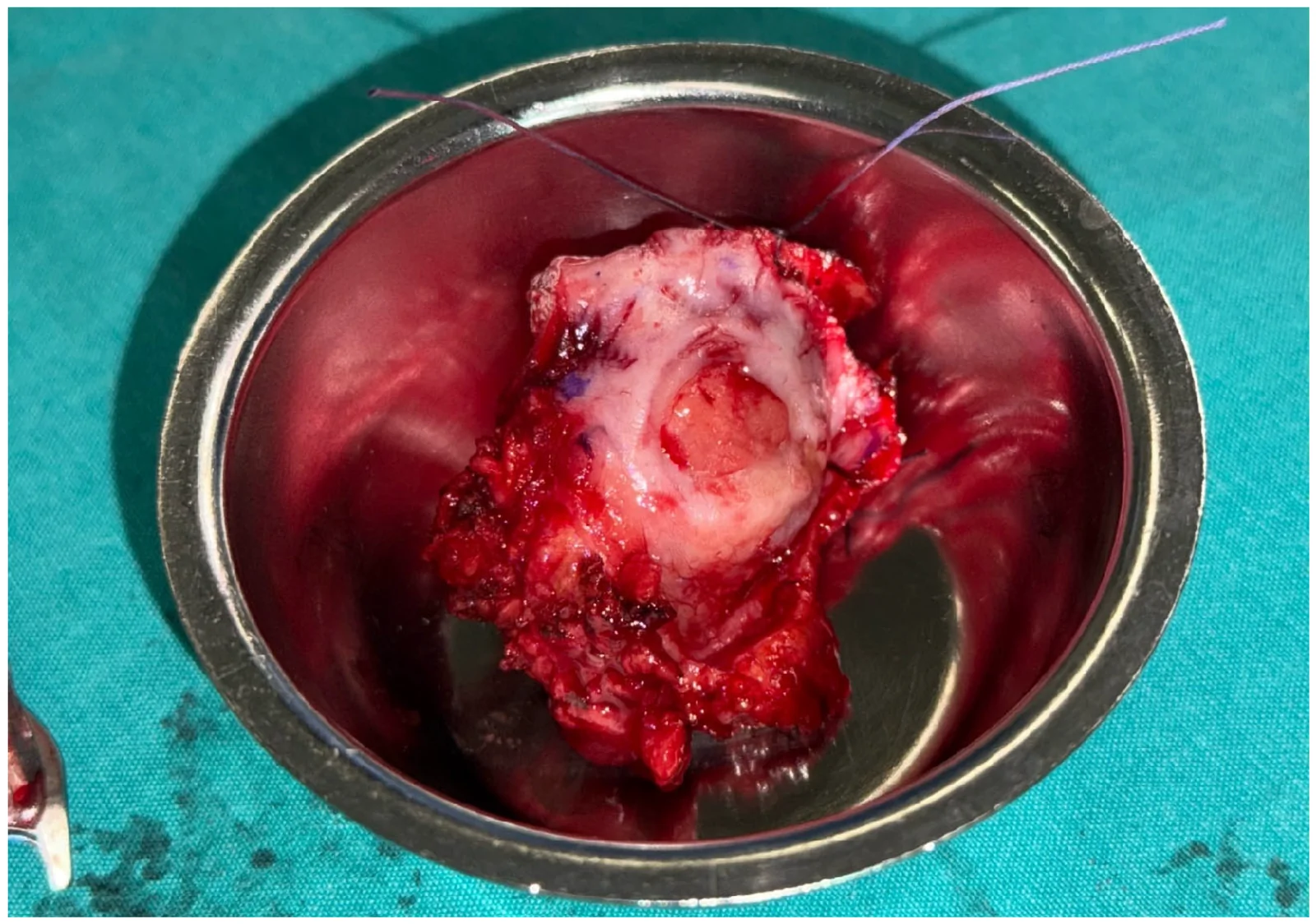
Oriented surgical specimen following wide local excision of the recurrent palatal lesion, including the surrounding mucosa and underlying periosteum.

**Figure 5:**
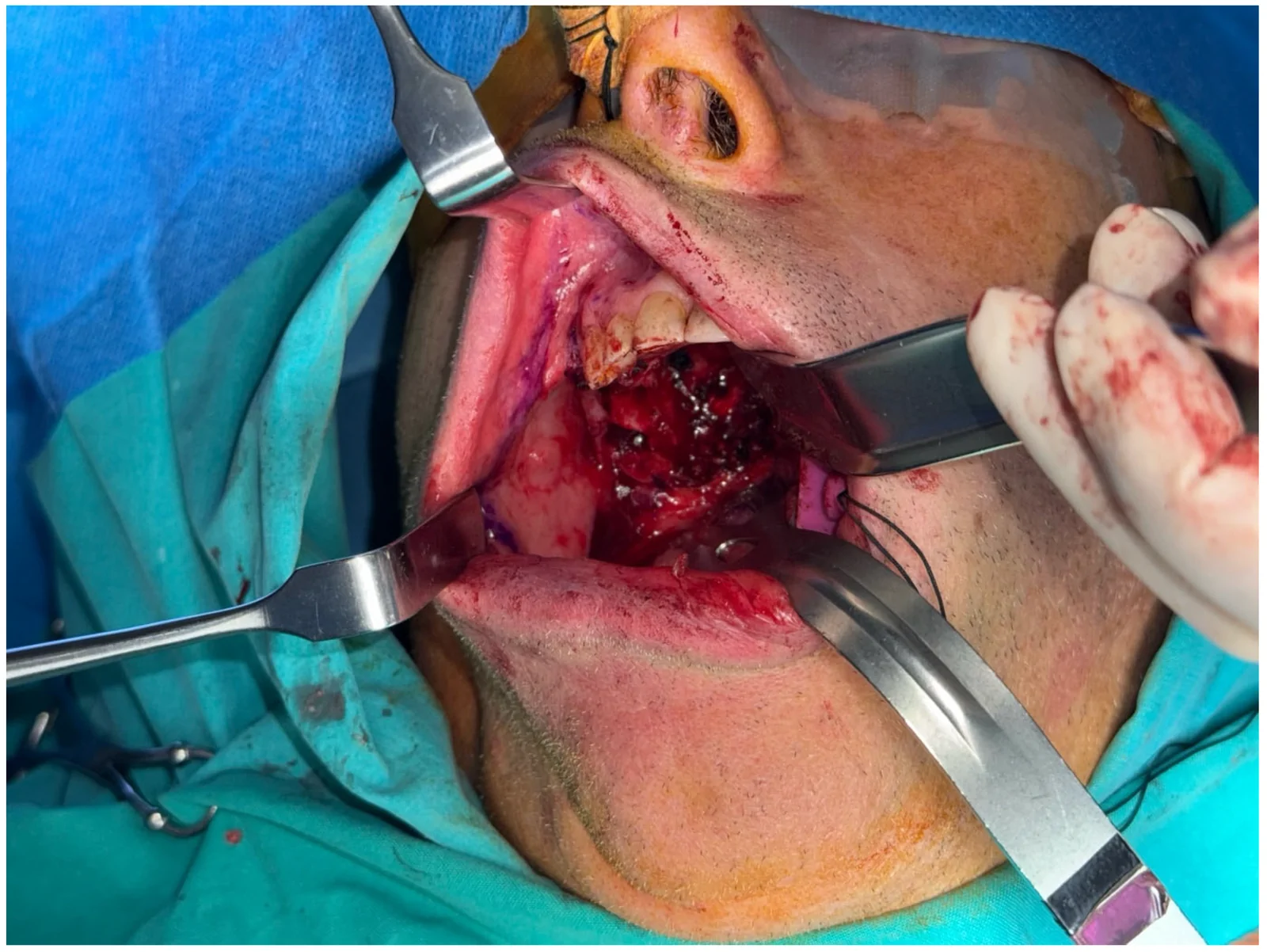
Intraoperative appearance of the palatal defect after complete tumor excision.

Histopathological examination of the incisional biopsy showed features consistent with pleomorphic adenoma, and definitive surgical treatment was planned. Under general anesthesia, the clinical limits of the tumor were marked before resection (Figure 3). Wide local excision was subsequently performed, including the lesion, surrounding palatal soft tissue, and underlying periosteum. The resected specimen contained the circumscribed tumor and was oriented with sutures for pathological assessment of the surgical margins (Figure 4). Intraoperative inspection demonstrated no macroscopic involvement of the adjacent maxillary bone or maxillary sinus (Figure 5).

**Figure 6:**
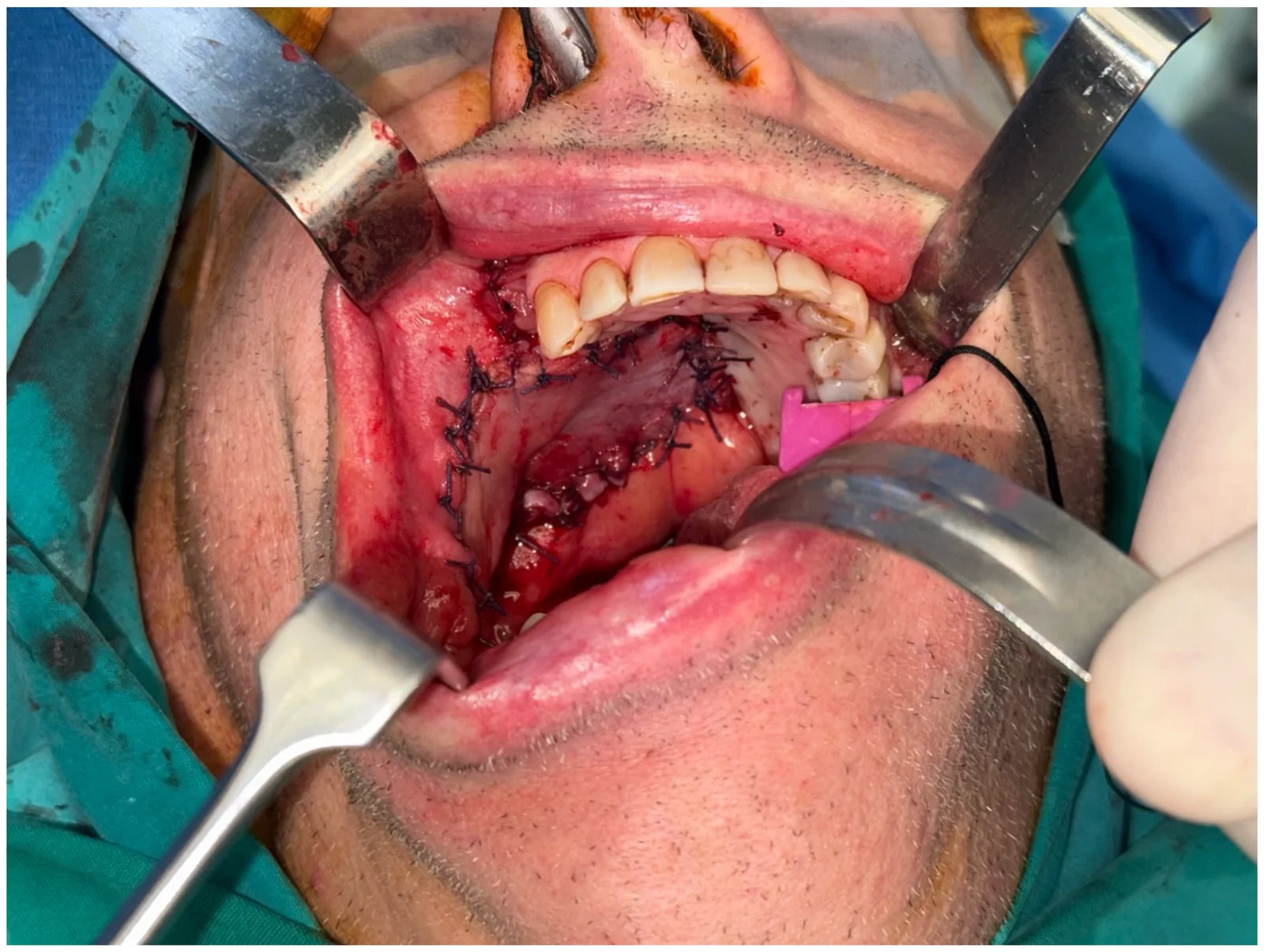
Final intraoperative appearance after reconstruction of the palatal soft-tissue defect with a facial artery musculomucosal flap, demonstrating satisfactory flap inset and coverage of the surgical defect.

The resulting palatal soft-tissue defect was reconstructed using a facial artery musculomucosal flap. Because the underlying palatal bone remained intact and there was no communication with the nasal or maxillary sinus cavities, the primary reconstructive objective was to provide well-vascularized mucosal coverage of the surgical defect rather than to separate the oral and sinonasal cavities. As the oncological resection included the underlying periosteum, an area of exposed palatal bone remained. The facial artery musculomucosal flap was selected because it provides thin, pliable, well-vascularized tissue with characteristics similar to the oral mucosa and offers an adequate arc of rotation for reconstruction of posterior palatal defects. In addition, its use avoided the need for a more extensive regional or free flap while allowing for immediate reconstruction of the defect. The flap was carefully elevated with preservation of its vascular supply, rotated into the palatal defect, and secured to the surrounding mucosal margins. The donor site was managed with primary mucosal closure where feasible (Figure 6).

Macroscopic pathological examination demonstrated an oriented surgical specimen measuring 4.0 × 3.2 × 2.3 cm. Its mucosal surface contained a central ulcerated area measuring approximately 1.2 × 1.1 cm, beneath which a well-circumscribed nodular lesion measuring approximately 2.0 × 1.7 × 2.2 cm was identified.

Histopathological examination confirmed recurrent pleomorphic adenoma of the minor salivary glands of the right palate. Surgical margins were clear, with no evidence of malignant transformation.

**Figure 7:**
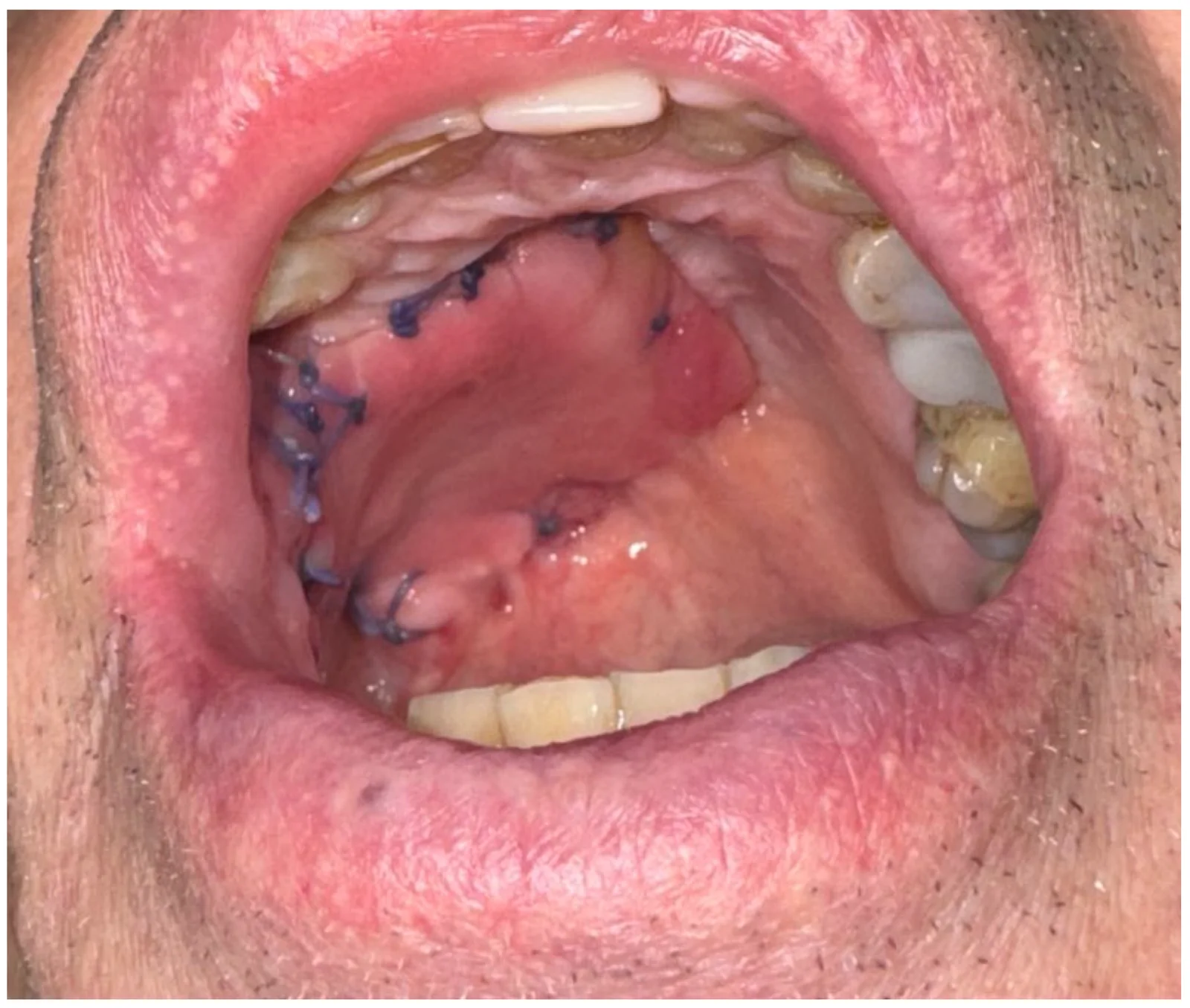
Fourteen-day postoperative follow-up showing satisfactory early healing of the palatal defect reconstructed with a facial artery musculomucosal flap.

The postoperative course was uneventful, with satisfactory flap healing and preservation of speech and swallowing. At the 14-day postoperative follow-up, the facial artery musculomucosal flap demonstrated satisfactory early healing (Figure 7). At the six-month follow-up, the reconstructed palate remained completely healed, without wound dehiscence, oroantral communication, clinically apparent recurrence, or functional impairment.

## 3. Discussion

Pleomorphic adenoma is the most common benign salivary-gland neoplasm and may arise from the minor salivary glands of the palate [1]. Although it is generally characterized by indolent biological behavior, local recurrence may occur, particularly following incomplete excision, capsular disruption, or persistence of microscopic tumor extensions beyond the apparent lesion boundaries [1,10].

The present patient developed a painless, slowly enlarging palatal recurrence approximately six years after the initial surgical treatment. This interval is consistent with the variable and sometimes prolonged pattern of recurrence reported for palatal pleomorphic adenoma. In the systematic review by Lopes-Santos et al. [10], recurrence was reported between 2 and 41 years after primary treatment, highlighting the potential for considerably delayed presentation. Comparable recurrent palatal lesions have been reported approximately 7.5 years after primary treatment by Hemavathy et al. [9] and after 10 years by De Lima et al. [4]. In both cases, complete surgical removal was emphasized as a central component of treatment.

The extent of recurrent disease may vary considerably. Nokaneng [12] described an invasive recurrent pleomorphic adenoma associated with osseous involvement, demonstrating that recurrent lesions may occasionally require more extensive surgical management. In contrast, the present case demonstrated no radiological or intraoperative evidence of maxillary bone involvement, allowing for treatment by wide local excision of the affected palatal soft tissues and underlying periosteum while preserving the osseous palate.

Very late recurrence remains an important characteristic of pleomorphic adenoma. Turner and Smith [5] reported recurrence 35 years after previous treatment, illustrating that a relatively short disease-free interval cannot reliably exclude subsequent recurrence. Consequently, prolonged clinical surveillance should be considered even after apparently complete excision.

Repeated recurrence and prolonged tumor duration are also clinically relevant because of the uncommon but recognized possibility of malignant transformation. Negahban et al. [11] described carcinoma arising in a long-standing recurrent pleomorphic adenoma of a minor salivary gland. In the present case, however, histological examination demonstrated no infiltrative growth, significant cytological atypia, prominent mitotic activity, conspicuous tumor necrosis, perineural invasion, lymphovascular invasion, or other morphological evidence of carcinoma ex pleomorphic adenoma.

Complete surgical excision with histologically negative margins remains the principal treatment for recurrent pleomorphic adenoma [4,10,12,13]. The extent of resection should be determined by the anatomical involvement of the recurrent tumor. Lesions confined to the palatal soft tissues without radiological or intraoperative evidence of bone involvement may be managed by wide soft-tissue excision, including the underlying periosteum, whereas lesions showing osseous invasion may require a more extensive maxillary resection [12,13].

In the present case, preservation of the underlying palatal bone resulted in a predominantly soft-tissue reconstructive defect. Reconstruction was considered appropriate to provide immediate vascularized mucosal coverage, promote wound healing, and preserve oral function. A facial artery musculomucosal flap was selected because it provides well-vascularized, pliable mucosal tissue with characteristics similar to those of the native oral mucosa and has a favorable arc of rotation for intraoral reconstruction [14]. The flap is particularly useful for selected small- to medium-sized oral cavity defects and may avoid the morbidity associated with more extensive reconstructive procedures [14]. In this patient, the flap provided satisfactory coverage of the posterior palatal defect and demonstrated uncomplicated healing during the early postoperative period.

At six months, the reconstructed palate remained clinically healed, without wound dehiscence or clinically apparent local recurrence, and the patient had no clinically evident impairment of speech or swallowing. However, this follow-up period is insufficient to establish durable disease control given the well-documented potential for recurrence many years after treatment [5,10]. Continued long-term surveillance is therefore warranted.

### Strengths and Limitations

A major strength of this case report is the detailed multidisciplinary documentation of an uncommon recurrent pleomorphic adenoma of the palate, including clinical examination, cross-sectional imaging, surgical management, and immediate reconstruction. The absence of radiological and intraoperative bone involvement permitted preservation of the hard-palate skeleton and reconstruction of the resulting soft-tissue defect using a facial artery musculomucosal flap. The case therefore provides clinically relevant information regarding both oncological management and reconstruction of a localized recurrent palatal lesion. Comparison with previously published cases further illustrates the heterogeneous presentation and extent of recurrent pleomorphic adenoma [4,5,9].

Several limitations should nevertheless be acknowledged. First, as a single-patient case report, the findings cannot be generalized to all patients with recurrent pleomorphic adenoma. Second, detailed information regarding the surgical technique used during the initial operation in 2020, including the status of the tumor capsule, possible intraoperative tumor spillage, and the precise histological margin status, was limited by the information available from the referring institution. Consequently, the exact mechanism responsible for recurrence cannot be established. Third, the postoperative follow-up period of six months is relatively short, particularly for pleomorphic adenoma, for which recurrence may become clinically apparent many years or even decades after initial treatment [4,10]. The absence of recurrence at six months should therefore be interpreted only as a favorable short-term outcome and not as evidence of definitive long-term disease control. Continued prolonged clinical surveillance is planned.

## 4. Conclusions

Recurrent pleomorphic adenoma of the palate is uncommon and may develop several years or even decades after initial treatment. In the present patient, a localized recurrence without osseous involvement was successfully managed by wide local excision with histologically negative margins followed by immediate reconstruction of the palatal soft-tissue defect using a facial artery musculomucosal flap. The reconstructive approach provided satisfactory short-term healing and preservation of oral function. However, the six-month follow-up period is insufficient to establish definitive long-term disease control. Given the potential for very late recurrence and the rare possibility of malignant transformation, prolonged clinical surveillance remains necessary.

## Data Availability

The original contributions presented in this study are included in the article/Supplementary Material. Further inquiries can be directed to the corresponding author.

## Supplementary Materials

The following supporting information can be downloaded at: https://www.mdpi.com/article/10.3390/clinpract16090168/s1, Table S1: CARE Checklist of information to include when writing a case report.

## References

[1] Menon G., Winters R. (2026). Pleomorphic Adenoma. StatPearls [Internet].

[2] Zar K., Tabib R., Rushinek H., Madmon I., Haran T.K., Alterman M. (2024). The correlation between histopathological pattern and surgical treatment for palatal pleomorphic adenoma. Can we choose a more conservative approach?. J. Cranio-Maxillofac. Surg..

[3] Lopes M.L.D.d.S., Barroso K.M.A., Henriques Á.C.G., dos Santos J.N., Martins M.D., de Souza L.B. (2017). Pleomorphic adenomas of the salivary glands: Retrospective multicentric study of 130 cases with emphasis on histopathological features. Eur. Arch. Oto-Rhino-Laryngol..

[4] De Lima F., Bezerra C., Rocha A., Martins I., Bernaola-Paredes W. (2020). Surgical Management of Palatal Pleomorphic Adenoma (PPA) Recurrence after 10 Years, Treated at a Brazilian Center—A Case Report. Ann. Maxillofac. Surg..

[5] Turner M.J.A., Smith W.P. (2006). An Exceptionally Late Recurrence of Pleomorphic Adenoma. J. Oral Maxillofac. Surg..

[6] Valstar M., de Ridder M., Broek E.v.D., Stuiver M., van Dijk B., van Velthuysen M., Balm A., Smeele L. (2017). Salivary gland pleomorphic adenoma in the Netherlands: A nationwide observational study of primary tumor incidence, malignant transformation, recurrence, and risk factors for recurrence. Oral Oncol..

[7] Choi S.Y., Choi J., Hwang I., Cho J., Ko Y.-H., Jeong H.-S. (2022). Comparative Longitudinal Analysis of Malignant Transformation in Pleomorphic Adenoma and Recurrent Pleomorphic Adenoma. J. Clin. Med..

[8] Moon S.Y. (2019). Surgical Management of the Palatal Pleomorphic Adenoma. J. Craniofacial Surg..

[9] Hemavathy K., Giri G.V.V., Subramani V., Susruthan M. (2022). Recurrent palatal pleomorphic adenoma: A case report with a long-term follow-up. Cureus.

[10] Lopes-Santos G., Marques N.G.D.O., Tjioe K.C., Oliveira D.T. (2024). Clinical Behavior of Recurrent Pleomorphic Adenoma in the Palate: A Systematic Review. Acta Cir. Bras..

[11] Negahban S., Daneshbod Y., Shishegar M. (2006). Clear Cell Carcinoma Arising from Pleomorphic Adenoma of a Minor Salivary Gland. Acta Cytol..

[12] Nokaneng E.N. (2022). Invasive Recurrent Pleomorphic Adenoma of the Palate: A Case Report and Literature Review. Oral Maxillofac. Surg. Cases.

[13] Ritwik P., Brannon R.B. (2012). A Clinical Analysis of Nine New Pediatric and Adolescent Cases of Benign Minor Salivary Gland Neoplasms and a Review of the Literature. J. Med. Case Rep..

[14] Khismatrao V.N., Popat S.P., Sharma P., Gupta A. (2023). Versatility of Facial Artery Musculomucosal (FAMM) Flap for Reconstruction of Oral Cavity Defects. J. Maxillofac. Oral Surg..

