# Supplementary material for: Recurrent Pleomorphic Adenoma of the Palate: A Case Report
Source: Clin Pract. 2026 Sep 10;16(9):168. doi: 10.3390/clinpract16090168 (PMC13605741; doi:10.3390/clinpract16090168)
Supplement: Supplementary file 1 [file clinpract-16-00168-s001.zip › clinpract-4532827-supplementary.pdf]

Table S1: CARE Checklist of information to include when writing a case report

Recurrent Pleomorphic Adenoma of the Palate: A Case Report

Topic	Item	Checklist item description	Reported on section / line
**Title**	**1**	The diagnosis or intervention of primary focus followed by the words 'case report'	Title - line 2
**Key Words**	**2**	2 to 5 key words that identify diagnoses or interventions in this case report, including 'case report'	Keywords, lines 38-39
**Abstract (no references)**	**3a**	Introduction: What is unique about this case and what does it add to the scientific literature?	Abstract - lines 14-18
	**3b**	Main symptoms and/or important clinical findings	Abstract - lines 19-23
	**3c**	The main diagnoses, therapeutic interventions, and outcomes	Abstract - lines 21-33
	**3d**	Conclusion - What is the main 'take-away' lesson(s) from this case?	Abstract - lines 33-37
**Introduction**	**4**	One or two paragraphs summarizing why this case is unique (may include references)	Introduction - lines 44-61
**Patient Information**	**5a**	De-identified patient-specific information	Case Presentation - lines 66-68
	**5b**	Primary concerns and symptoms of the patient	Case Presentation - lines 76-80
	**5c**	Medical, family, and psycho-social history including relevant genetic information	Case Presentation - lines 66-68
	**5d**	Relevant past interventions with outcomes	Case Presentation - lines 69-75
**Clinical Findings**	**6**	Describe significant physical examination (PE) and important clinical findings	Case Presentation - lines 78-80
**Timeline**	**7**	Historical and current information from this episode of care organized as a timeline	Case Presentation - lines 69-76, 81-109, and 160-164
**Diagnostic Assessment**	**8a**	Diagnostic testing (such as PE, laboratory testing, imaging, surveys)	Case Presentation - lines 78-86, 98-103, and 138-159
	**8b**	Diagnostic challenges (such as access to testing, financial, or cultural)	Not reported
	**8c**	Diagnosis (including other diagnoses considered)	Case Presentation - lines 103 and 154-159
	**8d**	Prognosis (such as staging in oncology) where applicable	Discussion - lines 217-221
**Therapeutic Intervention**	**9a**	Types of therapeutic intervention (such as pharmacologic, surgical, preventive, self-care)	Case Presentation - lines 103-106 and 122-132
	**9b**	Administration of therapeutic intervention (such as dosage, strength, duration)	Case Presentation - lines 103-109 and 122-132
	**9c**	Changes in therapeutic intervention (with rationale)	Not reported
**Follow-up and Outcomes**	**10a**	Clinician and patient-assessed outcomes (if available)	Case Presentation - lines 160-164, and Discussion - lines 217-219
	**10b**	Important follow-up diagnostic and other test results	Case Presentation - lines 160-164, and Discussion - lines 217-221
	**10c**	Intervention adherence and tolerability (How was this assessed?)	Not reported
	**10d**	Adverse and unanticipated events	Case Presentation - lines 160-164
**Discussion**	**11a**	A scientific discussion of the strengths AND limitations associated with this case report	Discussion - lines 222-242
	**11b**	Discussion of the relevant medical literature with references	Discussion - lines 171-221
	**11c**	The scientific rationale for any conclusions (including assessment of possible causes)	Discussion - lines 173-175, 184-221, and 232-242
	**11d**	The primary 'take-away' lessons of this case report (without references) in a one-paragraph conclusion	Conclusions - lines 244-252
**Patient Perspective**	**12**	The patient should share their perspective in one to two paragraphs on the treatment(s) they received	Not reported
**Informed Consent**	**13**	Did the patient give informed consent? Please provide if requested	Informed Consent Statement - lines 268-269
